# Recognizing the Importance of Dysphagia: Stumbling Blocks and Stepping Stones in the Twenty-First Century

**DOI:** 10.1007/s00455-016-9746-2

**Published:** 2016-08-29

**Authors:** Rainer Dziewas, Anne Marie Beck, Pere Clave, Shaheen Hamdy, Hans Jürgen Heppner, Susan E. Langmore, Andreas Leischker, Rosemary Martino, Petra Pluschinski, Andreas Roesler, Reza Shaker, Tobias Warnecke, Cornel Christian Sieber, Dorothee Volkert, Rainer Wirth

**Affiliations:** 1 Department of Neurology, University Hospital Münster, Albert-Schweitzer-Campus 1, 48149 Münster, Germany; 2 Institute of Nutrition and Midwifery, Metropolitan University College, Copenhagen, Denmark; 3 Centro de Investigación Biomédica en Red de Enfermedades Hepáticas y Digestivas (CIBERehd), Hospital de Mataró, Universitat Autònoma de Barcelona, Mataró, Spain; 4 Gastrointestinal Sciences, School of Medical Sciences, Faculty of Biology, Medicine and Health, Clinical Sciences Building, Salford Royal Hospital (Part of the Manchester Academic Health Sciences Centre (MAHSC)), University of Manchester, Eccles Old Road, Salford, M6 8HD UK; 5 Department of Geriatrics, Helios Klinikum Schwelm, Chair of Geriatrics of Witten-Herdecke University, Schwelm, Germany; 6 Speech Language Hearing Sciences & Otolaryngology, Boston University and Boston University School of Medicine, Boston, USA; 7 Department for Geriatrics, Alexianer Krefeld GmbH, Dießemer Bruch 81, 47805 Krefeld, Germany; 8 Department of Speech Language Pathology, University of Toronto, 160-500 University Avenue, Toronto, Canada; 9 Abteilung für Phoniatrie und Pädaudiologie, Universitätsklinikum Gießen und Marburg-Standort Marburg, Baldingerstraße 1, 35043 Marburg, Germany; 10 Katholisches Marienkrankenhaus GmbH, Alfredstraße 9, Hamburg, Germany; 11 Division of Gastroenterology and Hepatology, Medical College of Wisconsin, Milwaukee, Wisconsin USA; 12 Institute for Biomedicine of Aging, Friedrich-Alexander Universität Erlangen-Nürnberg, Kobergerstraße 60, Nürnberg, Germany; 13 Department of General Internal Medicine and Geriatrics, St. John of God Hospital Regensburg, Regensburg, Germany; 14 Department for Internal Medicine and Geriatrics, Marien-Hospital Borken, Borken, Germany

**Keywords:** Deglutition, Deglutition disorders, Aspiration pneumonia, Malnutrition, Dehydration, Presbyphagia

The oropharyngeal swallow involves a rapid, highly coordinated set of neuromuscular actions beginning with lip closure and terminating with opening of the upper esophageal sphincter. The central coordination of this complex sensorimotor task uses a widespread network of cortical, subcortical, and brainstem structures. Many diseases and disorders affecting the central swallowing network or downstream peripheral nerves, muscles, and structures may result in an impaired oropharyngeal swallow. In addition, aging is also associated with multifactorial changes of swallowing physiology for which the term presbyphagia has been coined. Oropharyngeal dysphagia broadly affects respiratory safety due to the increased risk of aspiration, and swallowing efficacy leading to the impeding danger of insufficient nutrition and hydration.

Both from the epidemiological and the individual patient perspective, the impact of dysphagia is considerable, and should be seen as a matter of serious concern. The true prevalence of oropharyngeal dysphagia is difficult to determine. Studies have presented prevalence figures in the context of the patient’s setting, the disease state, and country of interest. Specifically, in a large survey, swallowing problems were reported in approximately 1 % of children with nearly 18 % of them rating this impairment as “big” or “very big” [1]. At the other end of the age spectrum oropharyngeal dysphagia has been reported in about 10–27 % of older community dwelling residents [2–5]. In the nursing home, setting numbers are significantly higher and cross the 50 % margin, which is similar to figures reported for older individuals admitted to hospital with a diagnosis of pneumonia [6].

Disease-specific prevalence data for oropharyngeal dysphagia are also substantial. In particular, in preterm infants but also in typically developing children-feeding problems, otherwise unexplained respiratory symptoms and a failure to thrive are frequently caused by dysphagia and its sequelae [7, 8]. Furthermore, oropharyngeal dysphagia is reported in more than half of acute stroke patients and patients with traumatic brain injury, at least one-third of patients with Parkinson’s disease and dementia and a significant number of patients with neuromuscular disorders, such as amyotrophic lateral sclerosis and myasthenia gravis [9–14]. In view of the demographic shift, especially with increasing numbers of very old people, i.e., those aged ≥85 years, these already alarming figures will further increase in the coming years since many underlying pathologies, particularly stroke, dementia and Parkinson’s disease, are age related. In light of the increasing incidence of children with prematurity and medically complex conditions, this problem is also critically augmented at the other side of the age spectrum.

The clinical consequences of dysphagia are directly linked to the patient’s overall prognosis, and may include aspiration pneumonia, malnutrition, and dehydration. In the presence of disordered swallowing, mortality is increased and elevated rates of infectious complications have been reported for several medical conditions, such as stroke or Parkinson’s disease, but are also present in other patient populations [15]. Older patients discharged from general hospitals with both dysphagia and malnutrition presented a mortality rate of 65.8 % at 1-year follow-up [16]. In children, consequences of dysphagia may be particularly severe, since unlike adults they have rapidly developing physiological systems. In this changing state, even short-term problems with swallowing can interrupt normal development and cause serious long-term sequelae [17].

Apart from these hazardous physical ramifications, dysphagia has a significant impact on the psychological well-being of affected individuals and has been linked to low mood and depression [2]. Particularly in the elderly, social isolation is propagated by dysphagia, since individuals embarrassed by their swallowing difficulties and experiencing anxiety or panic during mealtimes tend to avoid eating in public [18].

Last but not least, dysphagia also puts a heavy burden on healthcare resources. It has been shown that stroke-related dysphagia increases post-stroke medical expenses by nearly 25 % [19]. In patients with Parkinson’s disease, the presence of gastrointestinal disorders—with dysphagia being one of the most prominent symptoms—caused an excess of healthcare costs of more than 10 % [20]. Well above both these numbers, dysphagia in Alzheimer disease has been linked to a 40 % increase in total healthcare expenditures [21]. Reasons for these dysphagia-driven cost increases are probably multifactorial. They may relate to an extended length of stay in hospital, higher rate of infectious complications, more frequent emergency room visits, larger proportion of patients discharged to rehabilitations settings and nursing homes, and higher costs for medical equipment and treatment [19].

Taken together, the physical, psychological, social, and financial burden of oropharyngeal dysphagia strongly impacts the patients, their caregivers, and the healthcare system that supports them. In sharp contrast to these multifaceted and often disastrous consequences, recognition of this symptom in the medical and political communities is strikingly poor and has not substantially changed since the first editorial was published in Dysphagia back in 1986 [22]. The following *stumbling blocks* may play a central role in the evolution of this collective neglect.

1. Professionals involved in dysphagia care-like speech language pathologists, specialist doctors, nurses and dietitians-usually care for an etiologically heterogeneous group of patients being hospitalized on different wards, departments, etc. of a given hospital. Therefore this symptom is generally not the focus of attention of the responsible, specialty-based medical ward staff.
2. Dysphagia experts are underrepresented in most hospitals and faced with overwhelming duties. Therefore, apart from providing dysphagia screening in a large number of patients in need, more sophisticated diagnostic assessment or implementation of different treatment modalities is often not feasible.
3. There are several well-established options to evaluate the act of swallowing, in particular the videofluoroscopic swallowing study, flexible endoscopic evaluation of swallowing and high-resolution manometry. However, due to insufficient resources, access to these tools remains limited, frequently compounding medical care and impeding meaningful research.
4. Possibly because of the multidisciplinary approach to dysphagia, nomenclature, diagnostic assessment and treatment selection in the area of deglutition disorders are often heterogeneous. This is detrimental to interprofessional communication, makes concerted efforts from the clinical, scientific, or political spectra difficult to realize, and represents a barrier to the interaction between clinicians and funding agencies.
5. The care for patients with swallowing disorders across the lifespan involves several different professions and requires substantial knowledge with regard to physiology, pathophysiology, diagnostic techniques and therapeutic options. Despite this, specific educational programs teaching all these aspects are uncommon.
6. Large funding programs tend to be disease specific. Dysphagia as a multi-etiological syndrome is rarely seen as the focus of attention. Large-scale clinical studies are therefore difficult to initiate. Linked to this point, dysphagia is also not close to the main interest of most clinical key opinion leaders.
7. Treatments for dysphagia are generally supported by low to moderate levels of evidence as compared to other medical diagnoses, such as cancer, coronary heart disease, or diabetes, to name just a few. Expert opinions and non-controlled observational studies prevail, while randomized controlled trials with meaningful clinical endpoints are rare. Therefore, given this lack of high-quality evidence, disease-specific guidelines often underrate or even neglect the issue of dysphagia.
8. A lack of engagement with big pharma and industry means that there is little or no incentive to drive innovation by scientists working in these sectors.
9. Due to the heterogeneous nature of underlying etiologies, there are few patient advocates and lobby groups who are driving the profile of dysphagia at a political level. Therefore, there is virtually no (ring-fenced) public funding of dysphagia-specific research.

In spite of these problems, difficulties, and shortcomings, there are also several *stepping stones* that may help to redress and improve matters in the years to come.

1. Dysphagia affects a vital human function and therefore is a crucial matter. The need for proper diagnostics and effective treatment strategies to preserve or re-establish a safe swallow is intuitively of high priority.
2. As stated above, care for patients with dysphagia is usually provided by a multidisciplinary team comprising speech language pathologists, physicians of different specialties, nurses and dietitians. These teams in principle present ideal “think tanks,” since problems are perceived from different professional perspectives, thereby enhancing creative generation of novel solutions and ideas.
3. Today, dysphagia is increasingly recognized as a holistic disorder. Adopting the ICF system endorsed by the WHO and the potential effects of dysphagia on its domains “health,” “functioning,” and “disability” has recently drawn new interest. This development will help to highlight the serious consequences of this disorder, including health economics, and makes them more comparable to those of other health conditions.
4. Remarkable progress in the area of structural and functional brain imaging as well as recent research in the field of neuroplasticity and motor learning have been instrumental to further explore and understand the complex architecture of the central swallowing network and its potential for reorganization and adaptation. Findings from these studies will help to elucidate the underlying pathophysiology of a variety of swallowing disorders and to adopt new treatment strategies.
5. Several new treatment strategies have been developed recently. In particular, different modalities of central and peripheral neurostimulation as well as pharmacological treatment options have appeared on the horizon and have already been tested in small randomized controlled trials. Encouraging results may drive a move towards greater utilization of these adjunctive or complimentary treatments for oropharyngeal dysphagia.

Taken together, oropharyngeal dysphagia is a highly relevant multi-etiological symptom slipping through the net of most healthcare systems worldwide. Knowledge and diagnostic methods have never been as good as today. The need for better patient care and scientific evidence is therefore a priority for the health care sector. Let us use this momentum through a greater collective and collaborative multidisciplinary effort to close the gap and achieve better treatment efficacy and quality of life for our patients.
